# New status of *Bichromomyia* subspecies (Diptera: Psychodidae: Phlebotominae) based on molecular taxonomy

**DOI:** 10.1093/jme/tjae099

**Published:** 2024-09-04

**Authors:** Yokomi N Lozano-Sardaneta, Herón Huerta, Yesenia Marquez-López, Atilano Contreras-Ramos

**Affiliations:** Colección Nacional de Insectos, Departamento de Zoología, Instituto de Biología, Universidad Nacional Autónoma de México, Ciudad de México, Mexico; Laboratorio de Entomología, Instituto de Diagnóstico y Referencia Epidemiológicos ‘Dr., Manuel Martínez Báez’, Ciudad de México, Mexico; Doctorado en Ciencias Biológicas y de la Salud, Universidad Autónoma Metropolitana, Ciudad de México, Mexico; Colección Nacional de Insectos, Departamento de Zoología, Instituto de Biología, Universidad Nacional Autónoma de México, Ciudad de México, Mexico

**Keywords:** sand fly, subspecies, COI, phylogeny, *flaviscutellata* complex

## Abstract

The sand fly of the genus *Bichromomyia* (Galati, 1995) includes 3 subspecies considered vectors of *Leishmania*, which share high morphological similarity. Through information from the Cytochrome Oxidase Subunit I (COI) gene, we provide complementary evidence to support that *Bichromomyia olmeca olmeca*, and *Bichromomyia olmeca bicolor*, should be raised to nominal species status. We recovered specimens of *Bi*. *o. olmeca* from Quintana Roo, Tabasco, and Oaxaca, Mexico, supply 17 new COI sequences, and also incorporate GenBank sequences for other *Bichromomyia* species. After a Maximum Likelihood (ML) analysis, all *Bichromomyia* species clustered with a bootstrap of 100%, although sequences of *Bichromomyia flaviscutellata* were divided into 2 clusters with an interspecific range distance of 11.16% between them, which confirm cryptic species in Brazil. The genetic distance of *Bi*. *o. olmeca* compared to related subspecies ranged between 12.59% and 14.64%. A total of 29 haplotypes (Hd = 0.987; π = 0.08783; S = 136) were recovered from the *Bichromomyia* sequences. Results of the TC network were consistent with the ML analysis, supporting that subspecies of *Bichromomyia* are genetically distinct and deserve being raised to valid species category: *Bichromomyia olmeca* (Vargas & Díaz-Nájera) and *Bichromomyia bicolor* (Fairchild & Theodor).

## Graphical Abstract

**Graphical Abstract:**
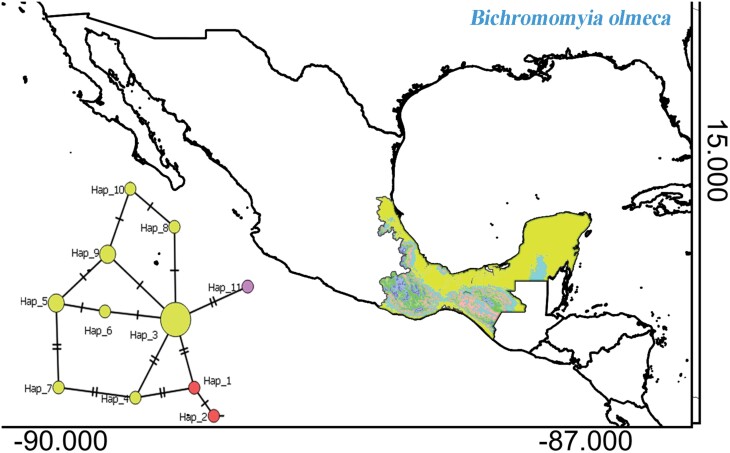

## Introduction

Phlebotomine sand flies are widely distributed in tropical and subtropical areas worldwide, where they relate with the transmission of the protozoan *Leishmania* (causal agent of leishmaniasis), the bacteria *Bartonella* and some *Phlebovirus*, a group of pathogens that affect millions of people and represents a serious public health problem (OMS 2020).

In the Americas, at least 555 sand fly species have been described, with only 98 of them relating to leishmaniasis transmission. *Bichromomyia* is one of the genera that encompasses species and subspecies of high relevance in the transmission of *Leishmania* spp. (Melo et al. 2020, Galati and Rodrigues 2023). One of such species groups, with large morphological similarity is the *flaviscutellata* complex: *Bichromomyia flaviscutellata sensu stricto* (*s*.*s*.), *Bichromomyia reducta*, *Bichromomyia inornata*, and 3 subspecies: *Bichromomyia olmeca olmeca*, *Bichromomyia olmeca bicolor*, and *Bichromomyia olmeca nociva* (Galati 2023).

In Mexico, *Bichromomyia olmeca olmeca* was originally described in 1959 from Teapa, Tabasco (*Phlebotomus olmecus*; *Lutzomyia* [*Nyssomyia*] *olmeca olmeca*; Vargas and Díaz-Nájera 1959, Young and Duncan 1994). Moreover, it was the first sand fly species incriminated as a vector of *Leishmania mexicana*, one of the causal agents of cutaneous leishmaniasis in humans and hamsters in Carrillo Puerto, Quintana Roo (Biagi et al. 1967), so it is considered one of the main leishmaniasis agent vectors in Mexico. This species is allopatric with regard to other members of the genus, which present a sympatric distribution (mainly in Brazil) (Melo et al. 2020). *Bichromomyia o. olmeca* has been recorded throughout southeastern Mexico, besides being known it is susceptible to disturbance and has preference for areas with high temperature and low precipitation, within an altitudinal range of 5–300 m above sea level (m.a.s.l.) (Montes de Oca-Aguilar et al. 2022, Lozano-Sardaneta et al. 2024a). Additionally, its geographic range extends to Guatemala, Belize, Honduras, Nicaragua, and Costa Rica (Shimabukuro et al. 2017).

Recently, molecular taxonomy using barcoding information suggested that the subspecies *Bi*. *olmeca nociva* and *Bi*. *olmeca bicolor* should be considered valid species based on genetic and phylogenetic analyses (Melo et al. 2020). However, *Bi*. *olmeca olmeca* had not yet been formally analyzed, although it was proven recently that this species and others members of the subgenus *Bichromomyia* displayed a high interspecific variability and different divergence times based on mitochondrial and nuclear genes (Lozano-Sardaneta, et al. 2023). Given that DNA barcodes using the Cytochrome Oxidase Subunit I (COI) gene have proven helpful in the molecular taxonomy of sand flies, allowing detection of cryptic species in different countries of the Americas (Melo et al. 2020, Lozano-Sardaneta et al. 2023), herein we aim to provide complementary evidence to validate that *Bi*. *o. olmeca* together with other subspecies of *Bichromomyia* should be raised to species category within the *flaviscutellata* complex.

## Materials and Methods

### Specimens Analyzed

*Bichromomyia o. olmeca* specimens were previously collected by Instituto de Diagnóstico y Referencia Epidemiológicos “Dr. Manuel Martínez Báez” (INDRE) of Secretaría de Salud, Mexico. The sampling localities were: 1) Huay Pix, Othón P. Blanco, Quintana Roo (18° 31ʹ 03.7″ N; 88° 25ʹ 24.6″ W; 10 m.a.s.l.), with sampling in December 2021 and July 2022; 2) El Zacatal, San Juan Guichicovi, Oaxaca (16° 56ʹ 29.0″ N; 95° 12ʹ 06.0″ W, 540 m.a.s.l.), July 2009; and 3) José María Pino Suárez, Cunduacán, Tabasco (18° 08ʹ 57.0″ N; 93° 17ʹ 30.0″ W; 10 m.a.s.l.), August 2009.

### Review of Geographical Distribution in Mexico

A database was created using Microsoft Excel^®^ with the bibliographic information from the distribution of *Bi*. *o*. *olmeca* in Mexico Vargas and Díaz-Nájera 1959, Biagi et al. 1967, Ibáñez-Bernal 2002, Godínez-Álvarez and Ibáñez-Bernal 2010, Sánchez-García et al. 2010, May-Uc et al. 2011, Moo-Llanes et al. 2013, Rodríguez-Rojas and Rebollar-Téllez 2017, Adeniran et al. 2019, Lozano-Sardaneta et al. 2020, 2022, 2024a, Montes de Oca-Aguilar et al. 2022, 2023, Cañeda-Guzmán et al. 2023, Martínez-Burgos et al. 2023). Considering the biogeographic provinces of Mexico (Morrone et al. 2017), a map of distribution was constructed with bibliographic data from specimens of *Bi*. *o*. *olmeca* using the free software QGIS (version 3.361).

### Morphological Identification

Using temporal mounting protocol (Lozano-Sardaneta et al. 2023), the taxonomic identification was performed using structures of the head and genitalia of males and females. The rest of the body was used for DNA extraction and PCR. Taxonomic classification was carried out following the proposal of Galati (2023). All specimens analyzed are housed at Colección Nacional de Insectos (CNIN), Instituto de Biología, UNAM.

### DNA Extraction, PCR, and Sequencing

DNA was extracted using Chelex-100 at 10%, following protocols previously standardized (Lozano-Sardaneta et al. 2021). The COI gene was amplified with the primers LCO1490 and HCO2198 to obtain a fragment of ~ 600 bp (Folmer et al. 1994). Protocols formerly standardized for PCR conditions were followed (Lozano-Sardaneta et al. 2021). PCR mixture was prepared at 25 μL with: 12.5 μL GoTaq Green Master Mix (Promega, Madison, USA), 1 μL of each primer (100 ng), 3–5 μL DNA (~60 ng/μL), and 7.5–5.5 μL nuclease-free water. Electrophoresis was performed in 1.5% agarose gel stained with 0.2 μL Midori Green Advance DNA stain (Nippon Genetics Europe). Purification and sequencing of PCR products took place at Laboratorio de Secuenciación Genómica de la Biodiversidad y de la Salud, Instituto de Biología, UNAM.

### Genetic and Phylogenetic Analysis

Chromas version 2.6.6 (http://technelysium.com.au/) was used for the visualization of electropherograms. Sequences were obtained from the NCBI database and compared using BLASTn (https://blast.ncbi.nlm.nih.gov/Blast.cgi). The accession numbers for the GenBank database are PP990478-PP990479 (for *Bi*. *olmeca* from Tabasco) and PP990480-PP990493 (for *Bi*. *olmeca* from Quintana Roo). The alignment was performed using the ClustalW algorithm in MEGA X (Kumar et al. 2018), including the new sequences and all the sequences available of the COI gene for the genus *Bichromomyia* from GenBank. Additionally, sequences of the sand fly subtribe Psychodopygina (genera *Nyssomyia* and *Psathyromyia*) available in GenBank were included.

The substitution model was selected considering the lowest Bayesian information criterion that showed a score of 6,155.003 to perform a Maximum Likelihood phylogenetic tree (ML), with 10,000 non-parametric bootstraps replicates using Tamura 3 parameters (T92) + Invariant sites (+I) substitution model in MEGA X. Genetic pairwise distances were calculated with Kimura-2-parameter substitution model (K2P) in MEGA X. The barcoding gap was obtained using Assemble Species by Automatic Partitioning (ASAP, https://bioinfo.mnhn.fr/abi/public/asap/) with Kimura (K80) ts/tv as substitution model (Puillandre et al. 2021).

The number of haplotypes (H), polymorphic sites (S), nucleotide diversity per species (π), haplotype diversity (Hd), number of haplotypes (Hn), and average number of nucleotide differences (K), was calculated using DnaSP v5.10 for performing the genetic analysis (Librado and Rozas 2009). Fixation index (*Fst*) was considered to differentiate the genetic structure of 3 species of the genus *Bichromomyia* using DnaSP v5.10 (Librado and Rozas 2009). PopART (http://popart.otago.ac.nz/) was used to construct a haplotype network graph, through TCS Networks and Minimum Spanning Networks to estimate gene genealogies (Bandelt et al. 1999, Clement et al. 2002).

## Results

### Species Collected and Their Distribution

**Fig 1.**
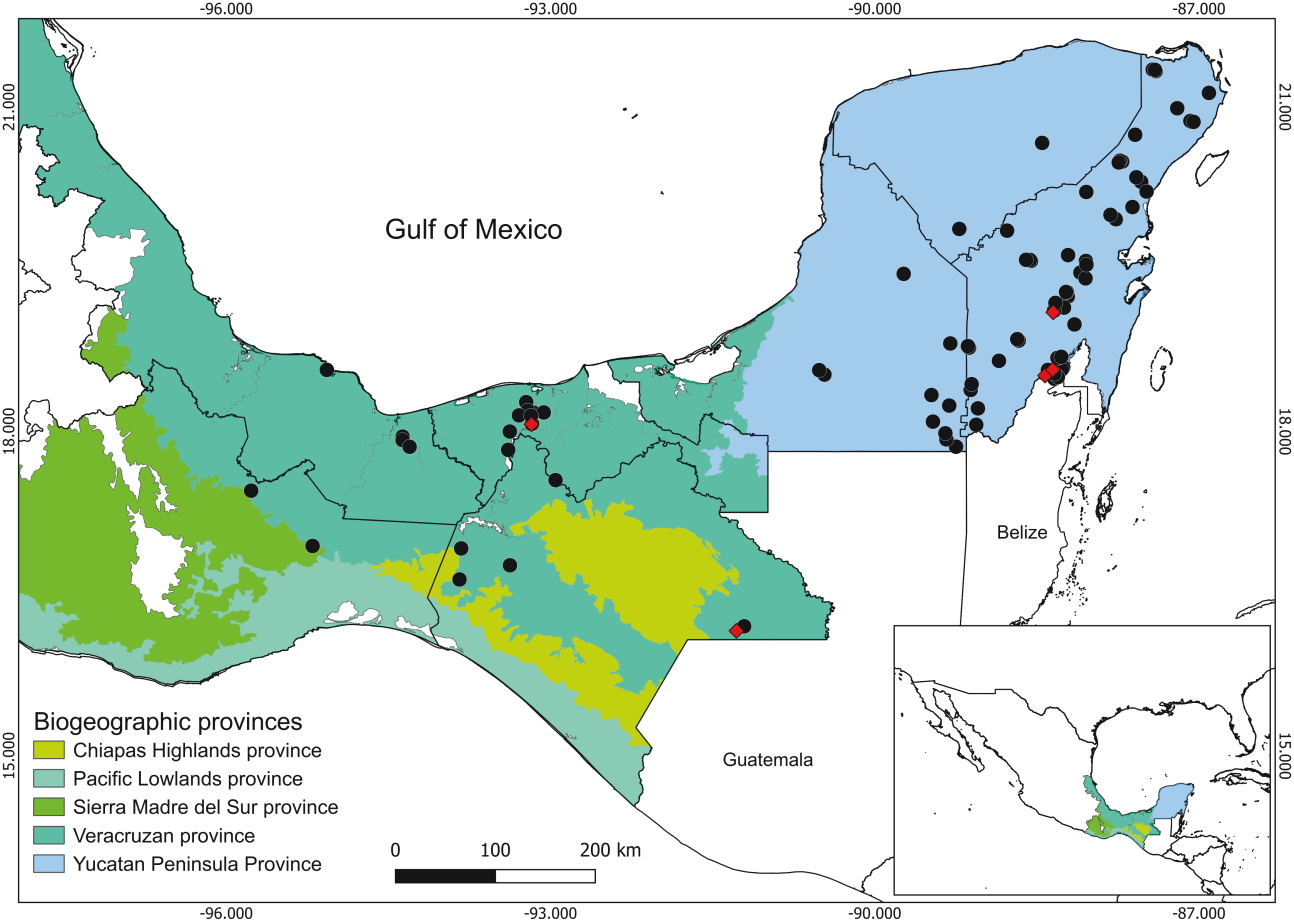
Distribution of *Bichromomyia olmeca olmeca* in Mexico according to biogeographic provinces. Circles represent historical distribution and diamonds represent sites with molecular information.

A total of 50 specimens of *Bi*. *o. olmeca* (2 ♂ and 48 ♀), from Quintana Roo (33 ♀; 1 ♂), Tabasco (16 ♀;1 ♂), and Oaxaca (1 ♀) were recovered. According to their historical distribution, 122 records were obtained in 64 localities from the states of Veracruz, Tabasco, Chiapas, Oaxaca, Campeche, Quintana Roo, and Yucatán. The highest number of occurrences was recorded in Quintana Roo (83/122), while Chiapas, Oaxaca, and Yucatán ranged between 2 and 3 records each (Fig. 1, Supplementary Fig. S1). Most of the *Bi*. *o*. *olmeca* records obtained, show that these insects are distributed mainly in areas below 500 m.a.s.l., which corresponds to plains and low-elevation mountains that belong to the Veracruzan and Yucatan Peninsula provinces (Fig. 1).

### Molecular Taxonomy and Phylogenetic Analysis

A total of 17 COI sequences from *Bi*. *o*. *olmeca* specimens were retrieved. A few specimens did not amplify (e.g., the specimen from Oaxaca), also a subsample from the specimens of Quintana Roo was chosen, since this was the state with the highest number of samples. Regarding COI sequences of *Bi*. *o*. *olmeca* available in GenBank, a similarity of 99% was determined with the sequence MK851274.1 from Othón Blanco, Quintana Roo, Mexico. Intraspecific variation in the genus *Bichromomyia* species ranged between 0.15% and 2.1%, being *Bi*. *flaviscutellata* (Brazil1) the species with the highest value (Table 1). Interspecific variability ranged between 12.04% and 16%, but some species recorded the highest genetic distances for instance *Bi. o*. *olmeca vs*. *Bi*. *o*. *bicolor* with 14.64%, *Bi*. *o*. *olmeca vs*. *Bi*. *flaviscutellata* (Brazil1) with 13.62%, *Bi*. *o*. *olmeca vs*. genus *Nyssomyia* with 13.43%; *Bi*. *o*. *bicolor vs*. *Nyssomyia* with 12.94%; *Bi*. *flaviscutellata vs*. *Nyssomyia* was 12.04%; *Nyssomyia vs*. *Psathyromyia shannoni* was 13.66%; and *Psathyromyia shannoni vs Bichromomyia* was 16% (Table 1).

**Table 1.** Genetic distances (Kimura 2-parameters) among sand fly species of the subtribe Psychodopygina

Genetic distances												
Species	[1]	[2]	[3]	[4]	[6]	[7]	[8]	[9]	[10]	[11]	[12]	Intraspecific variation
[1] *Bi*. *olmeca olmeca* [Tabasco]												0.17
[2] *Bi*. *olmeca olmeca* [Quintana Roo]	0.65											0–1.0
[3] *Bi*. *olmeca olmeca* [Chiapas]	1.72	0.98										—
[4] *Bi*. *olmeca bicolor* [Panama/Colombia]	14.53	15.12	14.27									0–0.69
[6] *Bi*. *flaviscutellata* [Brazil1]	12.84	13.52	14.52	12.24								0–2.1
[7] *Bi*. *flaviscutellata* [Brazil2]	11.89	12.46	13.43	13.26	11.16							0.15–1.4
[8] *Nyssomyia antunesi*	11.88	11.79	12.69	12.49	11.71	10.20						0.15
[9] *Nyssomyia umbratilis*	11.16	11.77	13.09	12.31	12.29	10.42	2.79					0.17–1.4
[10] *Nyssomyia whitmani*	14.09	14.01	15.31	13.25	13.72	12.10	4.51	5.36				0.62
[11] *Nyssomyia neivai*	14.72	14.74	16.00	13.71	13.70	12.18	4.81	5.10	1.94			0.64–0.81
[12] *Psathyromyia shannoni*	15.92	16.12	16.97	18.38	14.60	14.11	12.67	13.37	14.83	13.79		—

**Fig 2.**
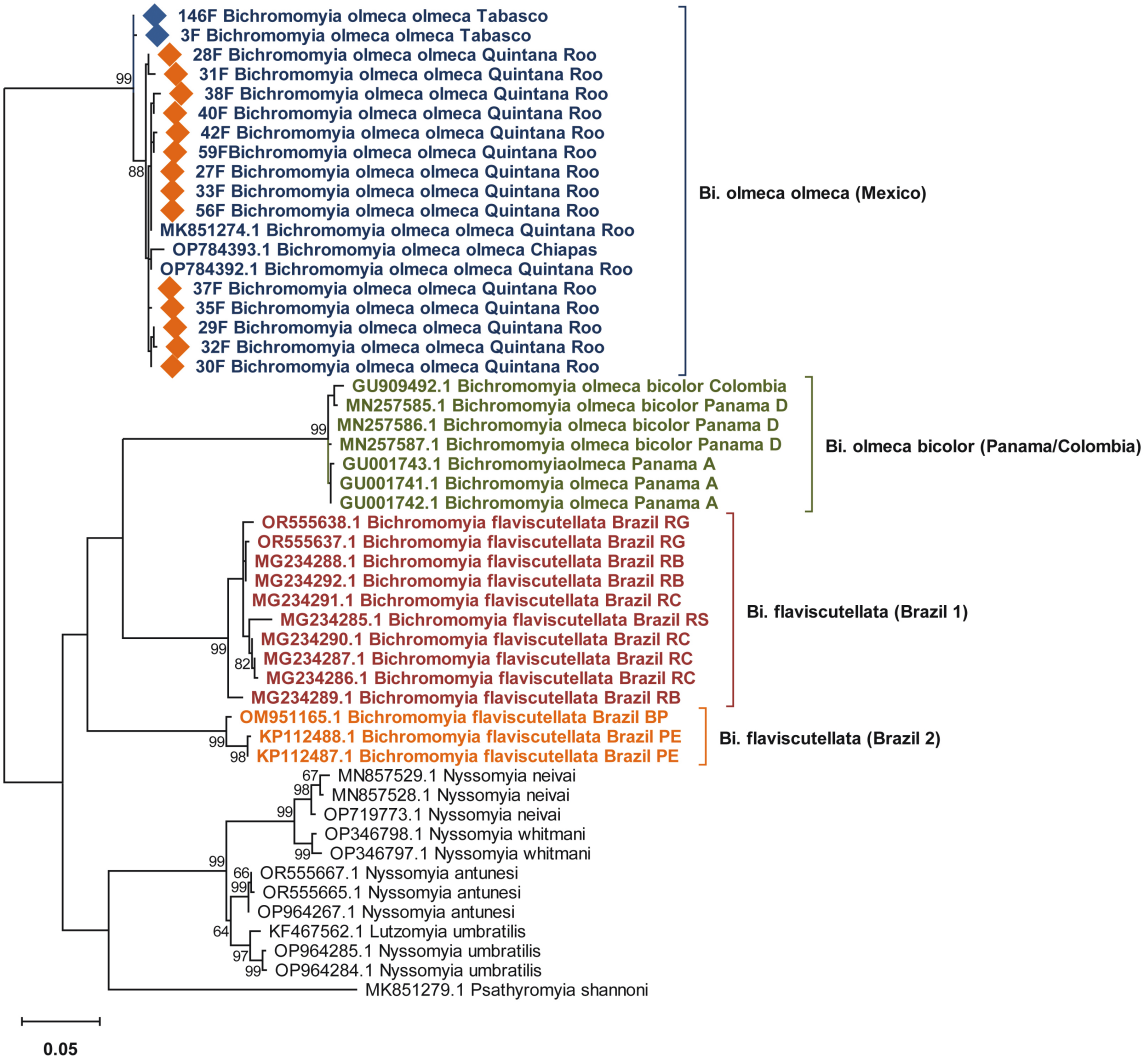
Phylogenetic relationships among 3 species of the genus *Bichromomyia* with respect to the genus *Nyssomyia* using a Maximum Likelihood analysis of the COI gene, with *Psathyromyia shannoni* as outgroup. Sequences from GenBank from previous studies of the genus *Bichromomyia* were utilized: Mexico (Adeniran et al. 2019, Lozano-Sardaneta et al. 2023); Panama PD = (Dutari and Loaiza 2019), PA = (Azpurua et al. 2010); Colombia CS = (Romero-Ricardo et al. 2016); Brazil RB = (Barreirinhas, Rodrigues et al. 2018), RC = (Colinas, Rodrigues et al. 2018), RS = (São Luís, Rodrigues et al. 2018), RG = (Maranhão, Governor Newton Bello, Rodrigues and Galati 2024), PE = (Espirito Santo, Pinto et al. 2015), BP = (Pernambuco, sequences directly submitted to GenBank OM951165.1, Baton & Shimabukuro, 2018), MN = (Novo Airão, Melo and Scarpassa 2024), MA = (Autazes, Melo and Scarpassa 2024). Diamonds highlight sequences generated in this study. Numbers in each node indicate bootstrap support.

The barcoding gap was calculated using only the molecular information of *Bichromomyia,* to obtain further evidence for species delimitation, the difference between interspecific and intraspecific distances did not show any overlap (Supplementary Fig. S2). ASAP partitions clustered the sequences analyzed into 4 subsets, with a best score showing a threshold distance of 7.2% under a *P*-value 1.42e-01, this result was consistent with the ML phylogenetic analysis (Fig. 2, Supplementary Fig. S3).

The alignment presented 649 sites, of which 466 were conserved sites and 183 were variable sites, the latter with 168 parsimony-informative sites, plus 15 singletons. The nucleotide diversity was π = 0.10228 and G + C = 0.341. No INDELs or stop codons were observed. In the ML tree, the sequences of the same species clustered with a bootstrap support of 100% (Fig. 2). *Bichromomyia o*. *olmeca* sequences from Quintana Roo and Tabasco, clustered with sequences from GenBank of the same species collected in Quintana Roo and Chiapas. Quintana Roo is the state with the most representative molecular information, although these sequences showed several singletons, their intraspecific variation (0%–0.78%) does not evidence that cryptic species are included.

The sequences labeled *Bi*. *olmeca* from Panama (Azpurua et al. 2010), clustered with sequences of *Bi*. *o*. *bicolor* from Colombia and Panama, confirming their identity as *Bi*. *o*. *bicolor*. Sequences of *Bi*. *flaviscutellata* available in GenBank, were divided into 2 groups, named in this work as Brazil1 (sequences from Barreirinhas, Colinas, São Luís, Maranhao, Novo Airão, and Autazes), and Brazil2 (sequences from Espirito Santo and Pernambuco; Fig. 2). These groups of sequences showed an interspecific distance of 11.16% between them. In addition, in the ML tree, they were not clustered as sister species, as sequences of Brazil1 that clustered with *Bi*. *o*. *bicolor* (Table 1, Fig. 2). The cluster of Brazil1 showed high intraspecific variability and the sequences were clustered in 4 genetic lineages (Fig. 2).

**Fig 3.**
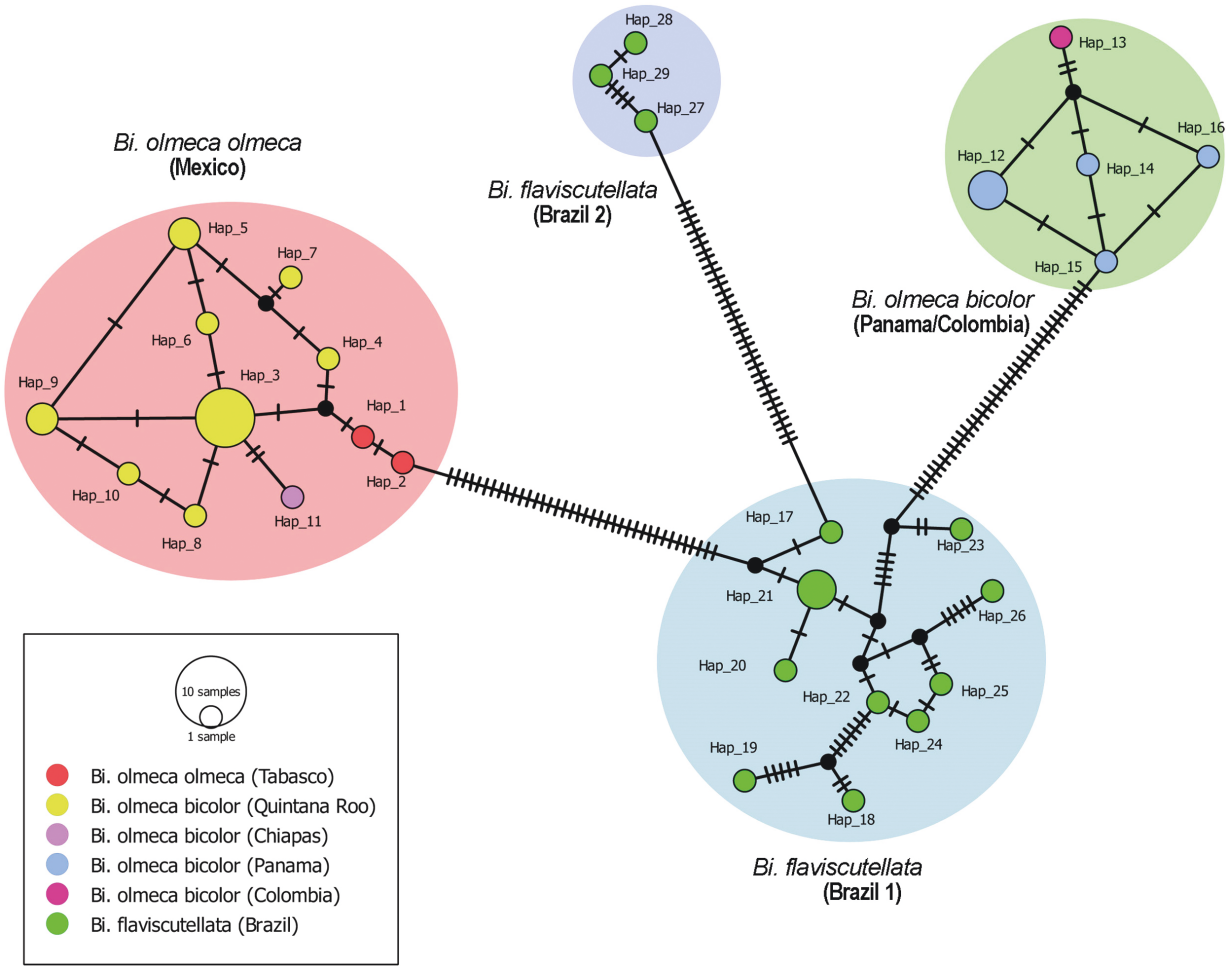
Haplotype TCS network of *Bichromomyia*. Circles highlight clusters obtained from each species; lines correspond to mutational steps; dots indicate missing haplotypes.

A total of 29 haplotypes (Hd = 0.987; π = 0.08783; S: 136) were obtained for the sequences of the genus *Bichromomyia*. The species *Bi*. *o*. *olmeca* recorded 11 haplotypes, 5 haplotypes for *Bi*. *o*. *bicolor* and 13 haplotypes for *Bi*. *flaviscutellata* [Brazil1/Brazil2] (Fig. 3, Table 2). The TCS network and the Minimum Spanning network (Supplementary Fig. S4), were consistent with the ML phylogenetic analysis, showing that species of *Bichromomyia* are genetically different since they are separated at least by 37 mutational steps (Fig 3; Supplementary Fig. S4). *Bichromomyia flaviscutellata* (Brazil1) and *Bi*. *o*. *olmeca* were the species with the highest haplotypes and nucleotide diversity (Fig. 3). Sequences of *Bi*. *flaviscutellata* became separated into 2 groups, hence it is likely that specimens of Brazil2 may, correspond to a separate species.

**Table 2.** Genetic differentiation between species of the genus *Bichromomyia*

Genetic differentiation							
Species	*n*	S	Hn	Hd	K	π	Haplotype frequency
*Bi*. *olmeca olmeca*	19	9	11	0.86550	2.07018	0.00641	H1 (1), H2 (1), H3 (7), H4 (1), H5 (2) H6 (1), H7 (1), H8 (1), H9 (2), H10 (1), H11 (1)
*Bi*. *olmeca bicolor*	7	4	5	0.85714	1.80952	0.00560	H12 (3), H13 (1), H14 (1), H15 (1) H16 (1)
*Bi*. *flaviscutellata* (Brazil1)	12	27	10	0.95455	7.72727	0.02392	H17 (1), H18 (1); H19 (1), H20 (1), H21 (3), H22 (1), H23 (1), H24 (1), H25 (1), H26 (1), H27 (1), H28 (1)
*Bi*. *flaviscutellata* (Brazil2)	3	5	3	1.00000	3.33333	0.01032	H27 (1), H28 (1); H29 (1)
Total	41	93	29	0.96463	33.03659	0.10228	

n, number of sequences; S, number of polymorphic/indel/missing sites; Hn, number of haplotypes; Hd, haplotype diversity; K, average number of nucleotide differences; π, nucleotide diversity; H1-2, Tabasco; H3, Quintana Roo, and sequences MK851274.1; OP784392.1; H4-10, Quintana Roo; H11, Chiapas OP784393.1; H12, Panama GU001741.1, GU001742.1, GU001743.1; H13, Colombia GU909492; H 14-16, Panama MN257585.1, MN257586.1, MN257587.1; H17-26, Brazil1 OR555638, OR511999.1, OR511998.1, OR555637.1, MG234292.1, MG234291.1, MG234288.1, MG234290.1, MG234289.1, MG234287.1 MG234286.1, and MG234285.1; H27-29, Brazil2 OM951165.1; KP112488.1; KP112487.1.

The values of fixation index *Fst* were *Bi*. *o*. *olmeca vs*. *Bi*. *o*. *bicolor *= 0.96; *Bi*. *o*. *olmeca vs*. *Bi*. *flaviscutellata* (Brazil1) = 0.90; *Bi*. *o*. *olmeca vs*. *Bi*. *flaviscutellata* (Brazil2) = 0.94; *Bi*. *o*. *bicolor vs*. *Bi*. *flaviscutellata* (Brazil1) = 0.90; *Bi*. *o*. *bicolor vs*. *Bi*. *flaviscutellata* (Brazil2) = 0.94; *Bi*. *flaviscutellata* (Brazil1 *vs*. Brazil2) = 0.86; and the global value was *Fst *= 0.95752. Because values for the 3 species are near to one, it may be supposed the genetic variation is explained by population structure, and that species do not share genetic diversity.

## Discussion

The significance of sand fly species falls mainly in their worldwide role as vectors of *Leishmania*. Therefore their correct systematic placement has great relevance, in order to optimize programs of control and prevention of vector-borne diseases. Until now, all species of the genus *Bichromomyia* encompass rodentophilic species that can feed also on human beings (Young and Duncan 1994, Melo and Scarpassa 2024), and may impact in relation to their capacity and competence for the transmission of several species of *Leishmania*, mainly species that cause cutaneous *Leishmania*sis in different Latin America countries (Melo et al. 2020).

The COI gene “barcode” (using the Folmer region) has been documented as a useful tool to establish taxonomic status and allows discrimination between close species (Hebert et al. 2003). Previous studies had already analyzed the molecular taxonomy of some species of *Bichromomyia* through 2 different COI regions [Folmer region (5ʹ) and Lunt region (3ʹ)] (Melo et al. 2020, Melo and Scarpassa 2024); however, in those studies sequences of the subspecies *Bi*. *o*. *olmeca* were not included. Therefore, information on *Bi*. *o*. *olmeca* is analyzed for the first time, in order to determine its genetic diversity and phylogenetic implication for its affinity with other *Bichromomyia* sand flies.

### Molecular Taxonomy and Phylogenetic Relationships Between *Bichromomyia* Sand Flies

Until now, sequence information on COI for the genus *Bichromomyia* was only available from Mexico, Panama, Colombia, and Brazil, with additional molecular studies missing, as the distribution of this genus covers several Latin American countries (Shimabukuro et al. 2017). According to the ML phylogenetic analysis, which incorporates novel data of *Bi*. *o*. *olmeca* and compares it to *Bichromomyia* information available in GenBank and BOLD, all species analyzed represent distinct genetic lineages with high bootstrap support, yet with a bootstrap value for this genus very low with respect to species of the genus *Nyssomyia*. This result has been recorded in other studies (Melo et al. 2020, Lozano-Sardaneta et al. 2023); however, it may improve if sequences from additional geographic areas, as well as from other species such as *Bichromomyia reducta* and *Bichromomyia inornata* (unsequenced species) are added, as genus monophyly has been validated with morphology (Galati 1995).

We confirm previous proposals that 3 subspecies (*Bi*. *o. nociva*, *Bi*. *o. bicolor*, and *Bi*. *o*. *olmeca*) placed in the genus *Bichromomyia* do each represent valid nominal species (Melo et al. 2020, Lozano-Sardaneta et al. 2023), and confirm a possible new species misidentified as *Bi*. *flaviscutellata* from Brazil. In the ML tree, the sequences of *Bi*. *o*. *bicolor*, from Panama and Colombia, clustered in the same clade, confirming that they belong to the same species. In GenBank, there are sequences (GU001741, GU001742, and GU001743) labeled *Bi*. *olmeca* from Panama, without specifying a subspecies (Azpurua et al. 2010). A previous analysis showed that such sequence clustered together with a sequence from Colombia (GU909492), with a low intraspecific distance, yet the authors concluded that corresponding subspecies (*Bi*. *o*. *bicolor* and *Bi*. *o*. *olmeca*) represent a single species (Melo et al. 2020). According to our results, sequences considered as *Bi*. *o*. *olmeca* from Panama (GU001741, GU001742, and GU001743), actually belong to subspecies *Bi*. *o*. *bicolor*, which is also consistent with the distribution recorded for *Bi*. *o*. *bicolor* (Brazil, Colombia, Costa Rica, Ecuador, Panama, Peru, Venezuela), meanwhile *Bi*. *o*. *olmeca* is only distributed from Mexico to Nicaragua (Shimabukuro et al. 2017). Therefore, these subspecies should not be considered the same entity. In the ML tree, *Bi*. *o*. *olmeca* represents a valid species that is separate from *Bi*. *o*. *bicolor* with an interspecific distance of 14.64%. Also, it has been proposed that the divergence time for *Bi*. *o*. *olmeca* from Mexico could have occurred 72.98 million years ago (mya), whereas *Bi*. *o*. *bicolor* from Colombia and Panama seems a species of recent evolutionary origin (15.7 mya), which probably diverged after the Isthmus of Panama formation, this suggests that *Bi*. *o*. *olmeca* might be endemic from the Veracruzan and Yucatan Peninsula provinces of Mexico (Lozano-Sardaneta, et al. 2023). Currently, only sequences from the states of Chiapas, Tabasco, and Quintana Roo in Mexico are available from *Bi*. *o*. *olmeca*; being Quintana Roo the best represented. Although the presence of cryptic species was discarded due to a low intraspecific variation, it is recommendable to incorporate additional molecular information of *Bi*. *o*. *olmeca*, from other states in Mexico, as well as from other countries (Belize, Costa Rica, Guatemala, Honduras, Nicaragua), to delimit their genetic diversity with respect to populations from Central America. Information from *Bi*. *o*. *nociva* was not available in the GenBank or BOLD Systems databases, even when sequences have been previously analyzed (Melo et al. 2020, Melo and Scarpassa 2024), however interspecific variability was recorded for this species with respect to *Bi*. *flaviscutellata s.s.* (12.7%) and *Bi*. *o. bicolor* (13%) (Melo et al. 2020), was similar to the values recorded in this study. Although formal comparison of interspecific variability between *Bi*. *o*. *nociva* and *Bi*. *o*. *olmeca* is still missing, the species *Bi*. *o*. *nociva* has only been recorded in Brazil and Peru (Shimabukuro et al. 2017), and the high interspecific variation between *Bi*. *o*. *nociva vs*. *Bi*. *flaviscutellata s.s.* and *Bi*. *o*. *bicolor* in Brazil, where they occur in sympatry, supports a hypothesis that *B. o. nociva* should be considered a valid species.

Here, all available sequences in GenBank of *Bi*. *flaviscutellata* generated from different studies in Brazil were analyzed, but clustered in 2 separate groups with an interspecific distance (K2P) of 11%. Recently, it has been confirmed that the species *Bi*. *flaviscutellata s*.*s*. showed a high haplotype diversity in Brazil (Melo and Scarpassa 2024), which is confirmed in the ML tree with the presence of at least 4 genetic lineages. The sequences of *Bi*. *flaviscutellata s*.*s*. (Brazil1), showed the highest nucleotide diversity, which confirm that it is a polymorphic species possibly in a process of diversification, which may correlate with reproductive isolation due to habitat fragmentation and the low flight capacity of sand flies (Melo et al. 2020, Melo and Scarpassa 2024). On the other hand, the sequences from Espirito Santo and Pernambuco, Brazil (labeled as Brazil2 in this work), confirm a cryptic species, results that coincide with a previous study (Rodrigues et al. 2018). According to our analysis, the cluster of Brazil2, is a sister species of *Bi. o. bicolor and Bi. flaviscutellata*, likely corresponding to a new species of *Bichromomyia* that would need an appropriate morphological and morphometric revision to elucidate its correct placement.

Potential reproductive isolation is supported, as species of *Bichromomyia* generally exhibit a discontinuous distribution, which appears to relate to the specific climatic and altitudinal requirements, besides changes in land use caused by human activities, contribute to limit their distribution and induce genetic differentiation (Valderrama et al. 2011, Montes de Oca-Aguilar et al. 2022, Lozano-Sardaneta et al. 2024a, Melo and Scarpassa 2024).

According to the haplotype network, we observed that all species analyzed are separated by several mutational steps (at least 37), with values of *Fst* near to one (value range of 0.89 to 0.96), which means such high differentiation in population structure supports our hypothesis that they are different species. Therefore, it is likely that geographical distribution and ecological components considerably influence genetic variation, favoring species divergence. Initially, it had been proposed that interspecific distance in insects should be >3% (Hebert et al. 2003). In sand flies it has been documented that intraspecific variation ranges from 0% to 6%, and the interspecific distance ranges from 9% to 26.7% (Contreras-Gutiérrez et al. 2014). Herein, intra- and interspecific ranges of variability were similar to other studies of sand flies, and values obtained fell within accepted limits to differentiate sand fly species, besides that the barcoding gap was high (7.2%) (Adeniran et al. 2019, Melo et al. 2020, Lozano-Sardaneta et al. 2023).

Further studies focusing on genetic structure to aid species delimitation in *Bichromomyia* are still needed, yet it is important to evaluate what region of the COI gene (Lut region or Folmer region) is a better tool to separate closely related species, in order to standardize amplification of the same region in all molecular studies, as these fragments only overlap across approximately 340 bp, which makes it difficult for information to be analyzed homogeneously.

### Morphological Evidence to Separate Bichromomyia Sand Flies

Besides phylogenetic and haplotype evidence, morphological studies include information for the correct separation of the subspecies *Bi. o*. *olmeca*, as displayed distinctive features allow separation of the species (Young and Duncan 1994, Galati 2023). In the case of males, differences are mainly found in the size of the 5th palpal segment compared to the third, the length of the epiandrial lobe compared to the gonocoxite, and the interocular distance (Galati 2023). Females show differences with respect to the number of posterior teeth (horizontal), which may vary between 8 and 12; length of the terminal knob, common duct, and individual ducts; as well as interocular distance (Galati 2023). Exochorion pattern also has been described as volcano-like with a rough appearance in the species *Bi*. *o*. *nociva* and *Bi*. *flaviscutellata s*.*s*. (Alencar and Scarpassa 2018). Meanwhile, *Bi*. *o*. *olmeca* shows a smaller egg size, with a layer with a polygonal pattern, and a second layer with a rough pattern similar to the volcano-like pattern (Lozano-Sardaneta et al. 2024b).

### Proposal for New Taxonomic Status of *Bichromomyia* Subspecies

Even though additional information on the genus *Bichromomyia* is needed, noteworthy evidence is provided that supports the 3 subspecies in the *flaviscutellata* complex should be considered under a valid species status, on the basis of molecular and morphological evidence as well as their distribution pattern. We herein propose these taxa be referred to as *Bichromomyia olmeca* (Vargas & Díaz-Nájera, 1959), new status; *Bichromomyia bicolor* (Fairchild & Theodor, 1971), new status, and *Bichromomyia nociva* (Young & Arias, 1982), new status. The last 2 species show a sympatric distribution with *Bi*. *flaviscutellata s*.*s*. in Brazil; however, the barcoding gap between these species, does not hinder validation of their species status (Melo et al. 2020).

## Supplementary data

Supplementary data are available at *Journal of Medical Entomology* online.

tjae099_suppl_Supplementary_Material
