# Supplementary material for: New status of Bichromomyia subspecies (Diptera: Psychodidae: Phlebotominae) based on molecular taxonomy
Source: J Med Entomol. 2024 Sep 4;61(6):1534–42. doi: 10.1093/jme/tjae099 (PMC11562967; doi:10.1093/jme/tjae099)
Supplement: tjae099_suppl_Supplementary_Material [file tjae099_suppl_supplementary_material.docx]

**New status of *Bichromomyia* subspecies (Diptera: Psychodidae: Phlebotominae) based on molecular taxonomy**

Yokomi N. Lozano-Sardaneta, Herón Huerta, Yesenia Marquez-López, Atilano Contreras-Ramos

**Supplementary table 1. Information of the geographic distribution of *Bichromomyia olmeca olmeca in* Mexico.**

| **Genus** | **Species** | **Locality** | **Latitude** | **Longitude** | **State** | **Country** |
| --- | --- | --- | --- | --- | --- | --- |
| *Bichromomyia* | *olmeca olmeca* | Altos de Sevilla | 18.8410 | -88.6671 | Quintana Roo | Mexico |
| *Bichromomyia* | *olmeca olmeca* | Altos de Sevilla | 18.8377 | -88.6681 | Quintana Roo | Mexico |
| *Bichromomyia* | *olmeca olmeca* | Andrés | 21.1322 | -86.9067 | Quintana Roo | Mexico |
| *Bichromomyia* | *olmeca olmeca* | Arroyo Negro | 17.8597 | -89.2472 | Campeche | Mexico |
| *Bichromomyia* | *olmeca olmeca* | Bencanche | 19.8742 | -89.2169 | Yucatán | Mexico |
| *Bichromomyia* | *olmeca olmeca* | Caobas | 18.3843 | -89.1105 | Quintana Roo | Mexico |
| *Bichromomyia* | *olmeca olmeca* | Caobas | 18.3846 | -89.1116 | Quintana Roo | Mexico |
| *Bichromomyia* | *olmeca olmeca* | Cárdenas | 18.0001 | -93.3764 | Tabasco | Mexico |
| *Bichromomyia* | *olmeca olmeca* | Central Vallarta | 20.8639 | -87.0661 | Quintana Roo | Mexico |
| *Bichromomyia* | *olmeca olmeca* | Central Vallarta | 20.8728 | -87.0784 | Quintana Roo | Mexico |
| *Bichromomyia* | *olmeca olmeca* | Chetumal - N1 | 18.5340 | -88.3279 | Quintana Roo | Mexico |
| *Bichromomyia* | *olmeca olmeca* | Chetumal - N10 | 18.5463 | -88.2983 | Quintana Roo | Mexico |
| *Bichromomyia* | *olmeca olmeca* | Chetumal - N11 | 18.5933 | -88.2556 | Quintana Roo | Mexico |
| *Bichromomyia* | *olmeca olmeca* | Chetumal - N12 | 18.5448 | -88.3890 | Quintana Roo | Mexico |
| *Bichromomyia* | *olmeca olmeca* | Chetumal - N2 | 18.5464 | -88.3228 | Quintana Roo | Mexico |
| *Bichromomyia* | *olmeca olmeca* | Chetumal - N3 | 18.5478 | -88.3238 | Quintana Roo | Mexico |
| *Bichromomyia* | *olmeca olmeca* | Chetumal - N4 | 18.5648 | -88.3161 | Quintana Roo | Mexico |
| *Bichromomyia* | *olmeca olmeca* | Chetumal - N5 | 18.5401 | -88.3216 | Quintana Roo | Mexico |
| *Bichromomyia* | *olmeca olmeca* | Chetumal - N6 | 18.5697 | -88.3967 | Quintana Roo | Mexico |
| *Bichromomyia* | *olmeca olmeca* | Chetumal - N7 | 18.5480 | -88.3217 | Quintana Roo | Mexico |
| *Bichromomyia* | *olmeca olmeca* | Chetumal - N9 | 18.5742 | -88.2686 | Quintana Roo | Mexico |
| *Bichromomyia* | *olmeca olmeca* | Chetumal - S1 | 18.5065 | -88.3229 | Quintana Roo | Mexico |
| *Bichromomyia* | *olmeca olmeca* | Chetumal - S2 | 18.5071 | -88.3228 | Quintana Roo | Mexico |
| *Bichromomyia* | *olmeca olmeca* | Chetumal - S3 | 18.5121 | -88.3228 | Quintana Roo | Mexico |
| *Bichromomyia* | *olmeca olmeca* | Chetumal - S4 | 18.4877 | -88.3380 | Quintana Roo | Mexico |
| *Bichromomyia* | *olmeca olmeca* | Chetumal - S5 | 18.5095 | -88.3472 | Quintana Roo | Mexico |
| *Bichromomyia* | *olmeca olmeca* | Chetumal - S6 | 18.4976 | -88.3366 | Quintana Roo | Mexico |
| *Bichromomyia* | *olmeca olmeca* | Chetumal - S7 | 18.4881 | -88.3360 | Quintana Roo | Mexico |
| *Bichromomyia* | *olmeca olmeca* | Chumpom | 19.9611 | -87.7661 | Quintana Roo | Mexico |
| *Bichromomyia* | *olmeca olmeca* | Chumpom | 19.9649 | -87.7702 | Quintana Roo | Mexico |
| *Bichromomyia* | *olmeca olmeca* | Chum-Yaxche | 20.0764 | -87.6144 | Quintana Roo | Mexico |
| *Bichromomyia* | *olmeca olmeca* | Chunhuas | 19.6319 | -88.2106 | Quintana Roo | Mexico |
| *Bichromomyia* | *olmeca olmeca* | Chunhuhub | 19.5783 | -88.5537 | Quintana Roo | Mexico |
| *Bichromomyia* | *olmeca olmeca* | Chunhuhub | 19.5889 | -88.5908 | Quintana Roo | Mexico |
| *Bichromomyia* | *olmeca olmeca* | Chunhuhub | 19.5872 | -88.5638 | Quintana Roo | Mexico |
| *Bichromomyia* | *olmeca olmeca* | Cinco de Mayo | 18.7759 | -89.1266 | Quintana Roo | Mexico |
| *Bichromomyia* | *olmeca olmeca* | Coba | 20.4967 | -87.7098 | Quintana Roo | Mexico |
| *Bichromomyia* | *olmeca olmeca* | Coba | 20.5046 | -87.7306 | Quintana Roo | Mexico |
| *Bichromomyia* | *olmeca olmeca* | Comalcalco | 18.2717 | -93.2247 | Tabasco | Mexico |
| *Bichromomyia* | *olmeca olmeca* | Coyolar | 17.9206 | -94.3689 | Veracruz | Mexico |
| *Bichromomyia* | *olmeca olmeca* | Cunduacán | 18.0672 | -93.1756 | Tabasco | Mexico |
| *Bichromomyia* | *olmeca olmeca* | Dos Lagunas Sur | 17.9275 | -89.3367 | Campeche | Mexico |
| *Bichromomyia* | *olmeca olmeca* | Dos Naciones | 17.9833 | -89.3457 | Campeche | Mexico |
| *Bichromomyia* | *olmeca olmeca* | Dzibalché | 19.4583 | -89.7314 | Campeche | Mexico |
| *Bichromomyia* | *olmeca olmeca* | Palenque | 16.6314 | -93.8419 | Chiapas | Mexico |
| *Bichromomyia* | *olmeca olmeca* | Emiliano Zapata | 17.9528 | -94.3686 | Veracruz | Mexico |
| *Bichromomyia* | *olmeca olmeca* | Felipe Carrillo Puerto | 19.5776 | -88.0454 | Quintana Roo | Mexico |
| *Bichromomyia* | *olmeca olmeca* | Francisco I. Madero | 20.2159 | -88.0419 | Quintana Roo | Mexico |
| *Bichromomyia* | *olmeca olmeca* | Francisco I. Madero | 18.1748 | -93.1589 | Tabasco | Mexico |
| *Bichromomyia* | *olmeca olmeca* | Huimanguillo | 17.8303 | -93.3914 | Tabasco | Mexico |
| *Bichromomyia* | *olmeca olmeca* | Jalpa de Méndez | 18.1764 | -93.0625 | Tabasco | Mexico |
| *Bichromomyia* | *olmeca olmeca* | La Guadalupe | 18.3373 | -89.4748 | Campeche | Mexico |
| *Bichromomyia* | *olmeca olmeca* | La Libertad | 18.5267 | -90.4648 | Campeche | Mexico |
| *Bichromomyia* | *olmeca olmeca* | La Libertad | 18.5675 | -90.5132 | Campeche | Mexico |
| *Bichromomyia* | *olmeca olmeca* | La Virgencita | 18.2397 | -89.3100 | Campeche | Mexico |
| *Bichromomyia* | *olmeca olmeca* | Laguna Guerrero | 18.6840 | -88.2982 | Quintana Roo | Mexico |
| *Bichromomyia* | *olmeca olmeca* | Laguna Guerrero | 18.6810 | -88.3087 | Quintana Roo | Mexico |
| *Bichromomyia* | *olmeca olmeca* | Lagunitas | 18.5036 | -88.3053 | Quintana Roo | Mexico |
| *Bichromomyia* | *olmeca olmeca* | Leona Vicario | 20.9903 | -87.2000 | Quintana Roo | Mexico |
| *Bichromomyia* | *olmeca olmeca* | Libertad | 18.0720 | -93.1713 | Tabasco | Mexico |
| *Bichromomyia* | *olmeca olmeca* | Macario Gómez | 20.3103 | -87.5381 | Quintana Roo | Mexico |
| *Bichromomyia* | *olmeca olmeca* | Macario Gómez | 20.3097 | -87.5337 | Quintana Roo | Mexico |
| *Bichromomyia* | *olmeca olmeca* | Veinte de Noviembre | 18.8144 | -89.3003 | Campeche | Mexico |
| *Bichromomyia* | *olmeca olmeca* | Nuevo Durango | 20.7452 | -87.5895 | Quintana Roo | Mexico |
| *Bichromomyia* | *olmeca olmeca* | Ocozocoautla | 16.7625 | -93.3750 | Chiapas | Mexico |
| *Bichromomyia* | *olmeca olmeca* | Once de Mayo | 18.0914 | -89.4608 | Campeche | Mexico |
| *Bichromomyia* | *olmeca olmeca* | Petcacab | 19.2488 | -88.2097 | Quintana Roo | Mexico |
| *Bichromomyia* | *olmeca olmeca* | Petcacab | 19.2517 | -88.2133 | Quintana Roo | Mexico |
| *Bichromomyia* | *olmeca olmeca* | Rancho La Ceiba | 19.8582 | -88.7748 | Quintana Roo | Mexico |
| *Bichromomyia* | *olmeca olmeca* | San Pedro Peralta | 18.6539 | -88.8499 | Quintana Roo | Mexico |
| *Bichromomyia* | *olmeca olmeca* | San Pedro Peralta | 18.6543 | -88.8476 | Quintana Roo | Mexico |
| *Bichromomyia* | *olmeca olmeca* | Santa Isabel | 19.4700 | -88.0961 | Quintana Roo | Mexico |
| *Bichromomyia* | *olmeca olmeca* | Santa María | 16.9186 | -93.8267 | Chiapas | Mexico |
| *Bichromomyia* | *olmeca olmeca* | Santiago Jalahui (Xalahui) | 17.4526 | -95.7701 | Oaxaca | Mexico |
| *Bichromomyia* | *olmeca olmeca* | Solferino | 21.3453 | -87.4062 | Quintana Roo | Mexico |
| *Bichromomyia* | *olmeca olmeca* | Solferino | 21.3342 | -87.3987 | Quintana Roo | Mexico |
| *Bichromomyia* | *olmeca olmeca* | Solidaridad | 18.5036 | -88.3053 | Quintana Roo | Mexico |
| *Bichromomyia* | *olmeca olmeca* | Teapa | 17.5505 | -92.9533 | Tabasco | Mexico |
| *Bichromomyia* | *olmeca olmeca* | Tomas Garrido | 18.0624 | -89.0603 | Quintana Roo | Mexico |
| *Bichromomyia* | *olmeca olmeca* | Tres Garantías | 21.3453 | -87.4062 | Quintana Roo | Mexico |
| *Bichromomyia* | *olmeca olmeca* | Tres Garantias | 18.2139 | -89.0445 | Quintana Roo | Mexico |
| *Bichromomyia* | *olmeca olmeca* | Uh-May | 19.4167 | -88.0486 | Quintana Roo | Mexico |
| *Bichromomyia* | *olmeca olmeca* | Veinte de Noviembre | 18.0914 | -89.4608 | Campeche | Mexico |
| *Bichromomyia* | *olmeca olmeca* | Zapotal | 17.8583 | -94.3036 | Veracruz | Mexico |
| *Bichromomyia* | *olmeca olmeca* | El Zacatal | 16.9414 | -95.2017 | Oaxaca | Mexico |
| *Bichromomyia* | *olmeca olmeca* | Huay Pix | 18.5177 | -88.4235 | Quintana Roo | Mexico |
| *Bichromomyia* | *olmeca olmeca* | Oriente 3ra sección | 18.1939 | -93.2114 | Tabasco | Mexico |
| *Bichromomyia* | *olmeca olmeca* | J. M. Pino Suárez | 18.1492 | -93.2917 | Tabasco | Mexico |
| *Bichromomyia* | *olmeca olmeca* | Altos de Sevilla | 18.8410 | -88.6671 | Quintana Roo | Mexico |
| *Bichromomyia* | *olmeca olmeca* | Huimango | 18.1461 | -93.1806 | Tabasco | Mexico |
| *Bichromomyia* | *olmeca olmeca* | Cunduacán | 18.0672 | -93.1756 | Tabasco | Mexico |
| *Bichromomyia* | *olmeca olmeca* | Finca Samuro | 17.5505 | -92.9533 | Tabasco | Mexico |
| *Bichromomyia* | *olmeca olmeca* | Chetumal | 18.5345 | -88.3353 | Quintana Roo | Mexico |
| *Bichromomyia* | *olmeca olmeca* | Felipe Carrillo Puerto | 19.5433 | -88.0403 | Quintana Roo | Mexico |
| *Bichromomyia* | *olmeca olmeca* | Aldea Tulum | 20.2198 | -87.4839 | Quintana Roo | Mexico |
| *Bichromomyia* | *olmeca olmeca* | Petcacab | 19.2901 | -88.2207 | Quintana Roo | Mexico |
| *Bichromomyia* | *olmeca olmeca* | Noh Bec | 19.1029 | -88.3476 | Quintana Roo | Mexico |
| *Bichromomyia* | *olmeca olmeca* | Noh Bec | 19.1463 | -88.2478 | Quintana Roo | Mexico |
| *Bichromomyia* | *olmeca olmeca* | Noh Bec | 19.1174 | -88.3408 | Quintana Roo | Mexico |
| *Bichromomyia* | *olmeca olmeca* | Noh Bec | 19.1896 | -88.3273 | Quintana Roo | Mexico |
| *Bichromomyia* | *olmeca olmeca* | Solferino | 21.3480 | -87.4269 | Quintana Roo | Mexico |
| *Bichromomyia* | *olmeca olmeca* | Central Vallarta | 20.8643 | -87.0493 | Quintana Roo | Mexico |
| *Bichromomyia* | *olmeca olmeca* | Cobá | 20.4887 | -87.7415 | Quintana Roo | Mexico |
| *Bichromomyia* | *olmeca olmeca* | Macario Gómez | 20.3517 | -87.5786 | Quintana Roo | Mexico |
| *Bichromomyia* | *olmeca olmeca* | Francisco I. Madero | 20.2159 | -88.0419 | Quintana Roo | Mexico |
| *Bichromomyia* | *olmeca olmeca* | Chumpon | 20.0042 | -87.8179 | Quintana Roo | Mexico |
| *Bichromomyia* | *olmeca olmeca* | Chunhuhub | 19.5883 | -88.5988 | Quintana Roo | Mexico |
| *Bichromomyia* | *olmeca olmeca* | Petcacab | 19.2908 | -88.2291 | Quintana Roo | Mexico |
| *Bichromomyia* | *olmeca olmeca* | Cinco de Mayo | 18.7896 | -89.1372 | Quintana Roo | Mexico |
| *Bichromomyia* | *olmeca olmeca* | Altos de Sevilla | 18.8541 | -88.6804 | Quintana Roo | Mexico |
| *Bichromomyia* | *olmeca olmeca* | San Pedro Peralta | 18.6539 | -88.8499 | Quintana Roo | Mexico |
| *Bichromomyia* | *olmeca olmeca* | Laguna Guerrero | 18.6905 | -88.2735 | Quintana Roo | Mexico |
| *Bichromomyia* | *olmeca olmeca* | Caobas | 18.4376 | -89.1030 | Quintana Roo | Mexico |
| *Bichromomyia* | *olmeca olmeca* | Tres Garantías | 21.3453 | -87.4062 | Quintana Roo | Mexico |
| *Bichromomyia* | *olmeca olmeca* | Limones | 18.9906 | -88.1511 | Quintana Roo | Mexico |
| *Bichromomyia* | *olmeca olmeca* | Los Tuxtlas | 18.5700 | -95.0700 | Veracruz | Mexico |
| *Bichromomyia* | *olmeca olmeca* | Coyolar | 17.9206 | -94.3689 | Veracruz | Mexico |
| *Bichromomyia* | *olmeca olmeca* | El Zapotal | 17.8583 | -94.3036 | Veracruz | Mexico |
| *Bichromomyia* | *olmeca olmeca* | Chetumal | 18.5719 | -88.3524 | Quintana Roo | Mexico |
| *Bichromomyia* | *olmeca olmeca* | Loop Xul | 20.6694 | -88.4506 | Yucatán | Mexico |
| *Bichromomyia* | *olmeca olmeca* | X-calaacop | 20.6508 | -88.5263 | Yucatán | Mexico |

**References**

Adeniran A, Fernández-Santos N, Rodríguez-Rojas J, et al (2019) Identification of phlebotomine sand flies (Diptera: Psychodidae) from leishmaniasis endemic areas in southeastern Mexico using DNA barcoding. Ecol Evol 9:13543–13554. https://doi.org/10.1002/ece3.5811

Biagi F, Biagi AMB, Beltrán F (1965) *Phlebotomus flaviscutellatus*, transmisor natural de Leishmania mexicana. Prensa Médica Mexexicana 30:267–272

Cañeda-Guzmán I, Montes de Oca-Aguilar A, Miranda-Caballero CI, et al (2023) Entomological survey and *Leishmania* (*Leishmania*) *mexicana* Prevalence in sand fly species during an outbreak of cutaneous leishmaniasis in Quintana Roo State, Mexico. Trop Med Infect Dis 8:. https://doi.org/10.3390/tropicalmed8100465

Godínez-Álvarez A, Ibáñez-Bernal S (2010) Catálogo de Psychodidae (Diptera) de la colección de artrópodos con importancia médica del InDRE, Secretaría de Salud, México. Acta Zoológica Mex 26:99–121

Ibáñez-Bernal S (2002) Phlebotominae (Diptera:Psychodidae) de México. III Las especies de *Lutzomyia* (*Psathyromyia*) Barreto, del grupo Aragoi, de *L*. (*Trichopygomyia*) Barreto, del grupo Dreisbachi y del *L*. (*Nyssomyia*) Barreto. Folia Entomológica Mex 41:149–183

Lozano-Sardaneta Y, Sánchez-Montes S, Sánchez-Cordero V, et al (2020) Molecular detection of *Leishmania infantum* in sand flies (Diptera: Psychodidae: Phlebotominae) from Veracruz, Mexico. Acta Trop 207:1–7. https://doi.org/https://doi.org/10.1016/j.actatropica.2020.105492

Lozano‑Sardaneta YN, Jacobo‑Olvera E, Ruiz‑Tovar K, et al (2022) Detection of *Wolbachia* and *Leishmania* DNA in sand flies (Diptera: Psychodidae, Phlebotominae) from a focus of cutaneous leishmaniasis in Tabasco, Mexico. Parasitol Res 121:513–520. https://doi.org/https://doi.org/10.1007/s00436-021-07412-4

Martínez-Burgos M, Lozano-Sardaneta YN, Rodríguez-Rojas JJ, et al (2023) Species diversity and detection of pathogens in phlebotomine sand flies collected from forest management areas of Quintana Roo, Mexico. Med Vet Entomol 37:845–858. https://doi.org/10.1111/mve.12691

May-Uc E, Hernández-Arana HA, Rebollar-Téllez EA (2011) Distribución de flebotominos (Diptera:Psychodidae) en Quintana Roo, México. Acta Zoológica Mex 27:273–289

Montes de Oca-Aguilar A, López-Ávila E, Sosa-Bibiano K, et al (2023) Registro de flebotominos antropofílicos en el peridomicilio de un caso de leishmaniosis cutánea localizada en Yucatán, México. Rev Biomédica 34:104–110. https://doi.org/https://doi.org/10.32776/revbiomed.v34i1.984

Montes de Oca-Aguilar A, Rebollar-Téllez EA, Sosa-Bibiano EI, et al (2022) Effect of land use change on the phlebotomine sand fly assemblages in an emergent focus of cutaneous leishmaniasis in Yucatan, Mexico. Acta Trop 235:106628. https://doi.org/https://doi.org/10.1016/j.actatropica.2022.106628

Moo-Llanes D, Ibarra-Cerdena CN, Rebollar-Téllez EA, et al (2013) Current and future niche of North and Central American sand flies (Diptera: Psychodidae) in climate change scenarios. PLoS Negl Trop Dis 7:e2421. https://doi.org/10.1371/journal.pntd.0002421

Rodríguez-Rojas JJ, Rebollar-Téllez EA (2017) Effect of trapping methods on the estimation of alpha diversity of a phlebotomine sandfly assemblage in southern Mexico. Med Vet Entomol 31:392–401. https://doi.org/10.1111/mve.12253

Sánchez-García L, Berzunza-Cruz M, Becker-Fauser I, Rebollar-Téllez EA (2010) Sand flies naturally infected by *Leishmania* (*L*.) *mexicana* in the peri-urban area of Chetumal city, Quintana Roo, Mexico. Trans R Soc Trop Med Hyg 104:406–411. https://doi.org/10.1016/j.trstmh.2010.01.010

Vargas L, Díaz-Najera A (1959) *Phlebotomus farilli* n.sp., *Ph. humboldti* n.sp. and *Ph. olmecus* n.sp. of Mexico (Diptera, Psychodidae). Rev Inst Salubr Enferm Trop 19:141–159

**
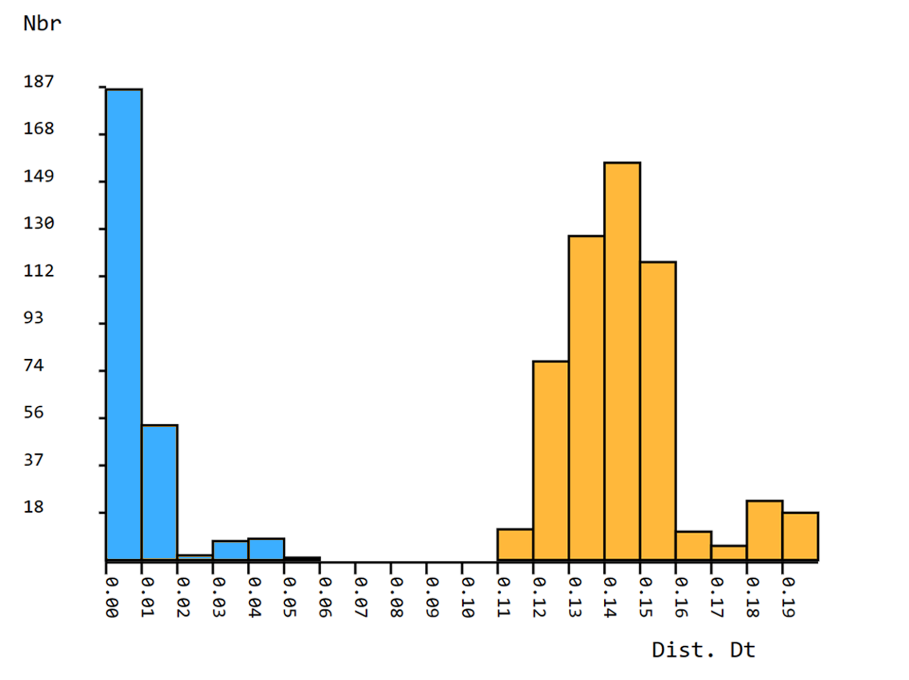
**

**Supplementary material 2. Histogram of intra- and interspecific genetic distances (barcoding gap) of *Bichromomyia* sand flies calculated with Kimura-2-parameter substitution model. Threshold distance was 7.2% (0.072354) with a *p* value 1.42e-01. Nbr = number; Dist. = distance value. Blue bars = intraspecific variation, and orange bars = interspecific variation.**


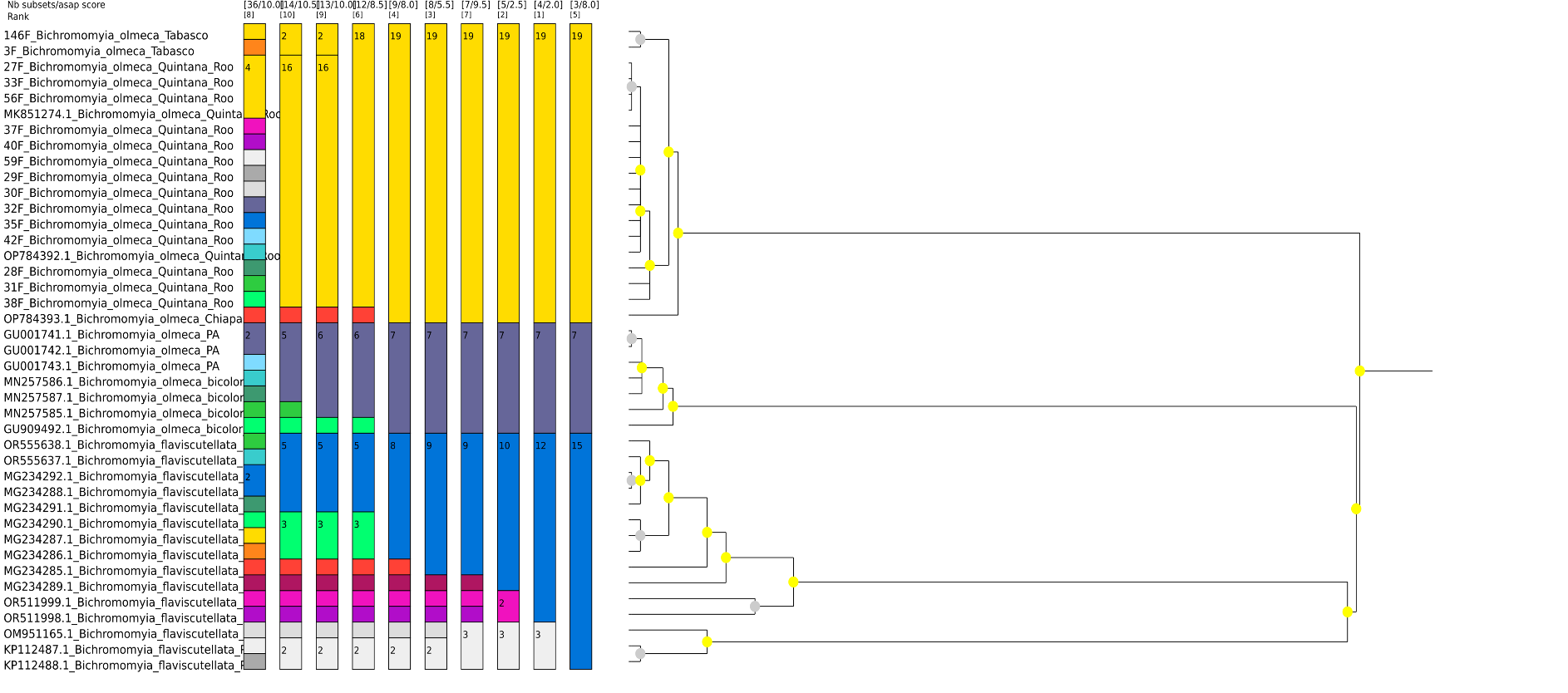


**Supplementary material 3. Ten ASAP partitions for clustering the sequences analyzed. The best score was four partitions showing a threshold distance of 7.2% under a *p* value 1.42e-01. The color of the bars shows the MOTUS, the number inside the bar represents the sequences number. The dendogram clustered the sequences according to their similarity.**


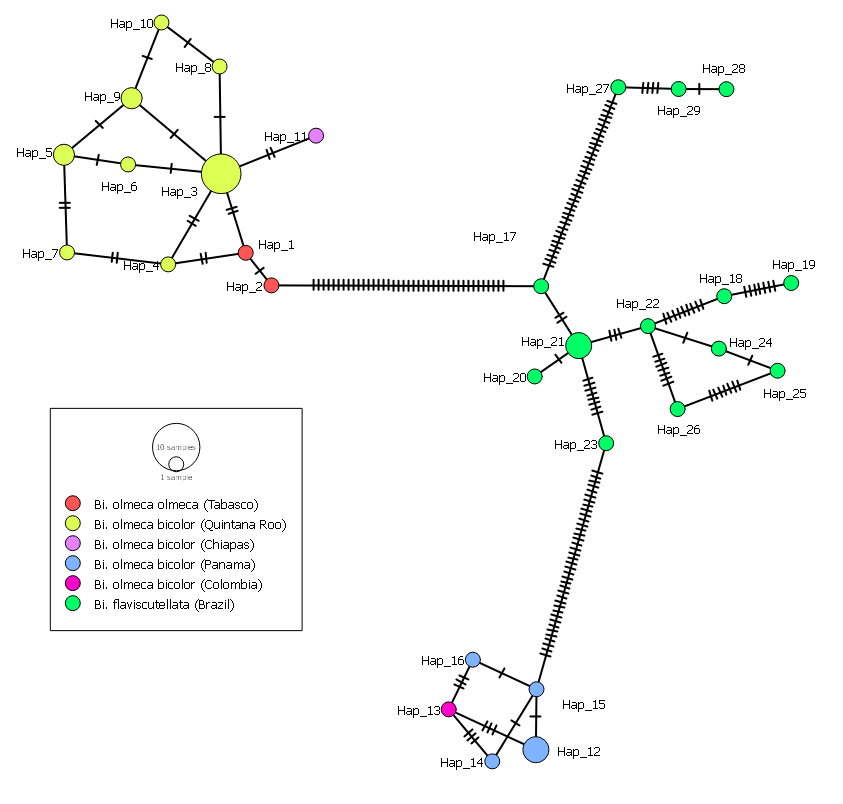


**Supplementary material 4. Haplotype Minimum Spanning Network of *Bichromomyia*. Black lines correspond to mutational steps; black dots indicate missing haplotypes.**
